# Minimum-Cost Offloading for Collaborative Task Execution of MEC-Assisted Platooning

**DOI:** 10.3390/s19040847

**Published:** 2019-02-18

**Authors:** Xiayan Fan, Taiping Cui, Chunyan Cao, Qianbin Chen, Kyung Sup Kwak

**Affiliations:** 1 School of Communication and Information Engineering, Chongqing University of Posts and Telecommunications, Chongqing 400065, China; Fanxiayan@yeah.net (X.F.); Caocyan@yeah.net (C.C.); chenqb@cqupt.edu.cn (Q.C.); 2 Chongqing Key Labs of Mobile Communications, Chongqing 400065, China; 3 School of Electrical and Computer Engineering, Inha University, Incheon 402-751, Korea; kskwak@inha.ac.kr

**Keywords:** platooning, collaborative task execution, mobile edge computing, offloading decision

## Abstract

In this paper, we study the offloading decision of collaborative task execution between platoon and Mobile Edge Computing (MEC) server. The mobile application is represented by a series of fine-grained tasks that form a linear topology, each of which is either executed on a local vehicle, offloaded to other members of the platoon, or offloaded to a MEC server. The objective of the design is to minimize the cost of tasks offloading and meets the deadline of tasks execution. The cost minimized task decision problem is transformed into the shortest path problem, which is limited by the deadline of the tasks on a directed acyclic graph. The classical Lagrangian Relaxation-based Aggregated Cost (LARAC) algorithm is adopted to solve the problem approximately. Numerical analysis shows that the scheduling method of the tasks decision can be well applied to the platoon scenario and execute the tasks in cooperation with the MEC server. In addition, compared with task local execution, platoon execution and MEC server execution, the optimal offloading decision for collaborative task execution can significantly reduce the cost of task execution and meet deadlines.

## 1. Introduction

With the explosive increase of vehicle terminals, new vehicular services such as 3D navigation, automatic driving and so on, which require the capability of supercomputing and mass storage, are developing rapidly. Recently, MEC technology has been proposed to solve the problem of global computing resource shortage caused by massive access of mobile devices in the fifth-generation mobile communication (5G) [1], which makes the transmission cost of mobile network lower and more efficient. MEC aims to reduce the pressure of vehicle terminals by offloading computing load to mobile edges. In the traditional wireless access network, the base station (BS) deployed on the edge of the mobile network carries out the services forwarding, but the BS does not actively analyze or respond to the user’s request. With the introduction of MEC, these MEC devices deployed on the edge of the network provide information technology service environment and cloud computing capability in the wireless access network nearest to user terminals (included vehicle terminals), e.g., small cell BS, macro cell BS or access to the MEC server by wireless access points, and minimize the transmission delay.

**Figure 1:**
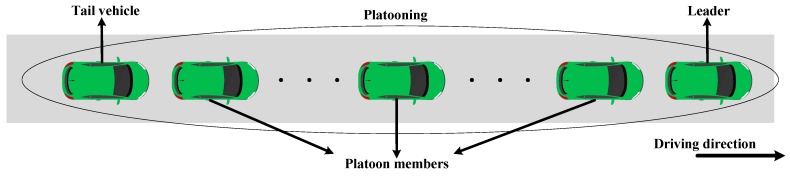
Platooning composition.

Platooning is the first step to realize automatic driving. It is the most representative case of 5G. In the platoon, the distance and speed between vehicles are controlled by automatic control system through real-time updated kinematics data [2]. Vehicles in a platoon are on the same driveway, like a train, and the distance between vehicles is fixed and very short. The higher frequency of vehicle information exchange in the platoon, the faster the maneuvering response of its members, and the more can avoid the unstable state of the platoon. This requires very high reliability and ultra-low delay data transmission, namely, vehicles should control the delay caused by the process of information processing and exchanging within 100 ms. On the other side, platooning is able to reduce fuel consumption and gas emission, as well as safe and efficient transportation in the context of intelligent transportation system (ITS) [3]. Generally, the platoon consists of two kinds of members: one is the leader (controller) and others are the members of the platoon (including the tail vehicle, the relay vehicle) [4], as shown in Figure 1. The longitudinal and lateral positions of each vehicle in the platoon are controlled by the collected data and vehicle status information (such as speed, acceleration, deceleration and location and so on). To reduce the possibility of the status of the platoon instability, these data should exchange frequently between vehicles in the platoon.

Most of the vehicle-related data are processed by the terminals that have been widely equipped on the vehicles [5]. More novel and attractive vehicle services have attracted more and more people to use them, such as 3D navigation, driverless, traffic information systems, voice processing and other vehicular networking applications. In the future, vehicles will be equipped with Augmented Reality (AR) applications that allow drivers to observe the surroundings of vehicles in windowless vehicles [6,7]. These types of vehicle applications are typical computing resource-hungry services, requiring high density computing resources and computing costs, and will be used to the platooning for better services. EyeDentify is a typical example [8], which matches the image based on target recognition algorithm. EyeDentify could be used as the security unlock for the vehicle users, and it includes a series of steps for feature extraction to transform the original image into a feature vector, and each of the step can be offloaded. Especially when running the delay-sensitive application on vehicle terminals, the vehicle users desire a fast response. For example, AR application combines computer-generated data with physical reality [9]. AR application contains five key components: the video source, the tracker, the mapper, the object recognizer and the renderer. First, the video sources must be collected by the local device. Second, the tracker, mapper and object recognizer are the computation-intensive components, which can be offloaded to the MEC server to reduce the computing time of the tasks and meet the task execution time requirements. Third, render must be executed locally and the calculated results need to be sent back to the local device for display. There are many relative applications for V2X services like Forward Collision Warning, Pre-crash Sensing Warning, V2X Road Safety Service via Infrastructure [10].

Due to the limited size of the resource space, the available resources of the vehicle terminals cannot meet the service requirements, which will cause high application processing delay, and even lead to safety accidents and so on. In addition, the current centralized server deployment in cellular network is not sufficient to meet future service requirements, such as delay, reliability and availability, which are key parameters. End-to-end delay must be reduced to within the range of service requirements (non-security services: 100 ms to 1 s, security related services or delay-sensitive services: less than 20 ms [7]). For instance, in current network architecture, offloading the workload of the vehicle terminal to the cloud center will cause extremely high transmission delay and it is difficult to achieve real-time data transmission [11]. The tension between computing resource-hungry applications and vehicle terminals with limited computing resources poses a major challenge to the development of mobile platforms [12]. In this paper, we consider a platoon with an MEC server to assist offload the workload of the vehicle terminal. Specifically, within the deadline of the application tasks, the resource purchase cost is employed to determine whether the tasks are executed locally, or offloaded to the other platoon members, or to the MEC server. We aim to find an optimal collaborative offloading decision among the members of the platoon and the MEC, and to execute the tasks with minimum resource purchase cost by the vehicle user within the deadline of the tasks. Mathematically, we model a minimum-cost task offloading problem as a constrained shortest path problem on a directed acyclic graph. Then we use the classical Lagrangian Relaxation-based Aggregated Cost (LARAC) algorithm to obtain an optimal solution of the constrained optimization problem. Finally, the simulation results of inputting the existing measurement data show that the offloading strategy is the optimal offloading strategy. Comparing the task execution locally or offloaded to the other members of the platoon, or offloaded the task to the MEC server, the cooperative task execution can complete the task within the task deadline at the minimum cost.

The rest of the paper is organized as follows. Section 2 reviews the relevant recent works. We give the system model in Section 3. In Section 4, delay constrained offloading decision is modeled as a constrained shortest path problem. Then we get an optimal strategy of offloading decision for collaborative task execution in Section 5. Section 6 shows the numerical analysis of offloading decision procedure, and Section 7 concludes the paper.

## 2. Related Work

There are a number of concepts that are closely related to MEC, for instance, mobile cloudlet systems, and MEC is considered to be the natural evolution of previous mobile cloud services (e.g., cloudlet) [13,14,15]. MEC provides a promising solution to the challenge for the development of mobile platforms and extends the capabilities of vehicle terminals, by providing additional computing, storage, and bandwidth resources in an on-demand manner [7].

MEC aims to further reduce delay, improve network operation efficiency, and promote service distribution capability, so as to improve the end-user experiences. Some of the proposed solutions can be applied to MEC scenarios. For example, an optimized structure for offloading a single mobile device to multiple MEC servers was proposed [16]. By jointly optimizing the task allocation decision and the Central Process Unit (CPU) frequency of the mobile device, the task execution delay and the energy consumption of the mobile device were minimized, but it only considered a single offloading architecture and could not achieve the multilayer tasks offloading. In addition, a game method could be used to solve the multi-user distributed computing offloading problem in multi-channel wireless interference environment, and provided users with computing resources by MEC at the edge of wireless access network [12]. However, the research did not consider the division of the application tasks, which were not flexible for execution of tasks. Some people considered offloading decision from the point of view of the servers, set the price for the unit resource provided to each vehicle user, and used the gain function of task offloaded to maximize the profit of the vehicular edge computing server [17], which increased the purchase cost of the vehicle user that was contrary to the interests of consumers. The authors of Reference [18] proposed an offloading algorithm minimized the completion time of the tasks. Some tasks were executed locally, and the other part were offloaded to the cloud. This algorithm could not be deployed directly to the realistic scenario, because reducing the completion time would result in too much processing cost. The authors of Reference [19] proposed a collaborative task execution strategy between the end-user and the cloud, which accomplished the tasks with minimum energy consumption within the application deadline. Its simulation results had proved that the collaborative task execution could significantly reduce the energy consumption on the mobile device, thus prolonged the lifetime of devices. The authors of Reference [20] used a directed graph to represent the code blocks and proposed a relatively genetic algorithm to determine how to offload the code blocks among multiple nodes. In Reference [21] the authors proposed an adaptive receding horizon offloading strategy, which could minimize the estimating cost of offloading among multiple devices and meet the time constraints of the tasks.

At present, there are lack of researches on tasks offloading in platoon, most of which are about connectivity [22,23] and communication strategy [24] of the network in which the platoon is located, but no consideration has been given to how to offload the tasks in the platoon. Due to the stability and predictability of platoon on the road [25], platoon members can also provide stable computing resources to users for a certain period of time, so as to obtain better computing performance and save the cost.

In order to have a deeper understanding of the offloading decision strategy, a simple task model is considered. Each task is executed one after another, forming a linear topology [19]. The task model is transformed into a constrained shortest path problem in directed acyclic graph. We deal with this problem under the path loss model and use Lagrange aggregation cost algorithm to obtain an approximate solution of the constrained problem. The main contributions of this article are summarized as follows:

- The optimization problem of the combined mobile application of each vehicle terminal in a platoon and an MEC server is modeled as a constrained minimizing cost problem.
- By analyzing the characteristics of task model in a linear topology, combining the dynamic programming algorithm and the Lagrangian aggregation cost algorithm, the optimal task decision strategy is obtained under the condition that the deadline of the tasks is satisfied.
- The effectiveness of the proposed algorithm and strategy is verified by simulation. The simulation results show that, in comparison with task platoon execution and the MEC server execution, collaborative task execution can greatly reduce the cost of tasks offloading.

## 3. System Model

**Figure 2:**
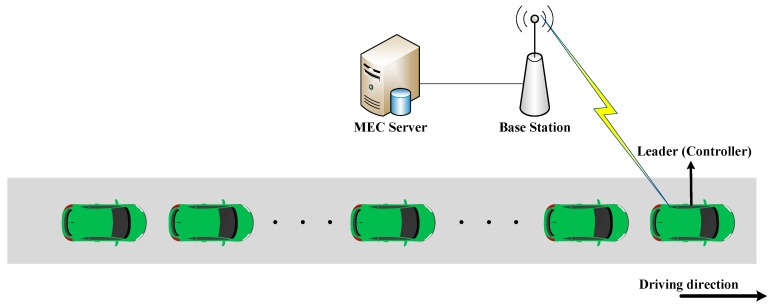
An MEC-assisted platooning scenario.

Figure 2 depicts a MEC server assisted platooning in our paper. The platoon controller controls and manages the whole platoon. When the platoon members communicate with the BS, they need to transmit the messages to the BS through the platoon controller, and the members in the platoon can directly communicate with each other [25,26]. Position of the controller in the platoon is not clearly defined in the current research. Most of the articles directly select the leader as platoon controller for the sake of simplification. In this paper, we do not discuss in detail how to choose the controller, which can be selected according to the signal intensity, platoon length and channel multiplexing.

We next introduce the system model of the optimal offloading decision for collaborative task execution, including task model, path loss model and task execution model.

### 3.1. Task Model

**Figure 3:**
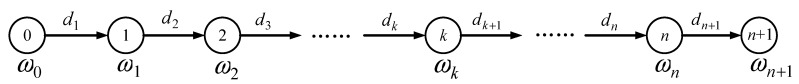
Task model in a linear topology.

Figure 3 illustrates a sequence of tasks with a linear topology, in the granularity of either a method or a module [19]. Each task is executed in sequence, the output of the previous task is the input of the later task, and the whole application has a completion deadline $T_{d}$. Note that there are n+2 tasks in an application. Define the demanded computation cycles of the task k as $\omega_{k}$, and the input data size of task k as $d_{k}$, k=0, 1, 2, …, n+1. According to the paper [27], the computing resources of demand have the following relationship with the size of the input data, $\omega_{k}=\phi d_{k}$, where ϕ is the computational complexity of the task. The value of ϕ depends on the nature of the task, which is beyond the scope of this paper. The following assumptions are adopted to model the practical problem of tasks. First, the output of a task must be replicated to the other platoon members or the MEC server before the next task is executed. Second, the first and last task of the application must be executed in the same vehicle in the platooning.

### 3.2. Path Loss Model

In the paper, we consider task offloading for MEC-assisted platooning in a wireless cellular network. The communication mode of platoon members is Vehicle to Vehicle (V2V) communication, which is deployed according to IEEE 802.11p standard [28]. The communication mode between vehicle and BS is Vehicle to Infrastructure (V2I) communication, which is deployed according to LTE-V [29].

**Figure 4:**
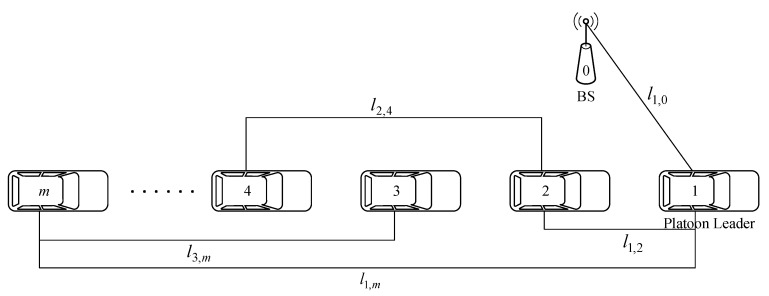
Illustration of the distance of platooning and base station (BS).

When one vehicle is served by another, the path loss ${\mathrm{PL}}_{v}\left(l_{v_{1},v_{2}}\right)$ between the vehicle $v_{1}$ and vehicle $v_{2}$ is modeled by Reference [30]

(1) $${\mathrm{PL}}_{v}\left(l_{v_{1},v_{2}}\right)=63.3+17.7\log_{10}\left(l_{v_{1},v_{2}}\right)$$

where $l_{v_{1},v_{2}}$ is distance in kilometers between two vehicles. Additionally, the path loss ${\mathrm{PL}}_{\mathrm{MEC}}\left(l_{q,u}\right)$ between the leader and BS is modeled by Reference [31]

(2) $${\mathrm{PL}}_{\mathrm{MEC}}\left(l_{q,u}\right)=128.1+37.5\log_{10}\left(l_{q,u}\right)$$

where $l_{q,u}$ is the distance in kilometers between the leader and BS, q=1, u=0 or q=0, u=1. Here, for convenience of discussion, we do not consider fast fading and shadow fading in our wireless communication model. The carrier frequency used in V2I communication is 2 GHz and V2V communication is 5.9 GHz. Therefore, there is no interference between V2I and V2V communication. The distances between vehicles in the platoon and the distances between the platoon and BS are illustrated in Figure 4. The MEC server is numbered 0, the platoon leader vehicle is numbered 1, and the sequence number of vehicles following it increases in turn. $l_{v_{1},v_{2}}$ is the geographic distance from $v_{1}$ to $v_{2}$, where $v_{1}$ and $v_{2}$ are the vehicle terminal numbers, $v_{1},\;v_{2}=1,\;2,\;\ldots ,\;m$ and $v_{1}\neq v_{2}$.

Considering that under ideal conditions all the vehicles in the platoon are of the same length and the same spacing so that the distance between the signal transmitter ($v_{1}$) and the receiver ($v_{2}$) in the platoon is the length of the vehicle plus the vehicle spacing. Then we get

(3) $$l_{v_{1},v_{2}}=\mu \left|v_{1}-v_{2}\right|$$

where μ is the distance in kilometers between the adjacent transmitter and the receiver. $\left|v_{1}-v_{2}\right|$ is the number of intervals. Specially, considering offloading tasks to the MEC server, and the distance in kilometers between the leader and the BS is set to η, we obtain

(4) $$l_{1,0}=\eta .$$

When the task is offloaded to the MEC server, the change of distance between the leader and the BS has negligible effect on the path loss, so we get $l_{1,0}=l_{0,1}$. 

### 3.3. Task Execution Model

In task execution, the models of platoon execution, MEC server execution, platoon data transmission, and MEC server data transmission are considered in our paper.

#### 3.3.1. Platoon Execution

The application can be initiated by any member in the platoon. The platoon execution includes local execution and platoon members execution. Every member in the platoon has the opportunity to participate in the execution of the tasks. If the task k is offloaded to $v_{2}$, the computing time of the task k is expressed by

(5) $$t_{k}^{v_{2}}=\frac{\omega_{k}}{f_{v_{2}}}$$

and the cost of offloading the task k is

(6) $$b_{k}^{v_{2}}=\alpha_{v}f_{v_{2}}$$

where $f_{v_{2}}$ is the computation resource that the vehicle $v_{2}$ can provide and $\alpha_{v}$ is the computation resource cost of each unit provided by the platoon members. Suppose that the unit price of resources provided by each member is the same, $f_{v_{2}}$ is constant during the task execution.

#### 3.3.2. MEC Server Execution

If the task k is executed on the MEC server (numbered 0), the computing time of the task k is expressed by

(7) $$t_{k}^{0}=\frac{\omega_{k}}{f_{0}}$$

and the cost of offloading the task k is

(8) $$b_{k}^{0}=\alpha_{\mathrm{MEC}}f_{0}$$

where $f_{0}$ is the computation resource that the MEC server can provide and $\alpha_{\mathrm{MEC}}$ is the computation resource cost of each unit provided by the MEC server. Note that the MEC server can provide more computation resources than platoon members, i.e., $f_{0}>f_{1},\;f_{0}>f_{2},\;\ldots ,\;f_{0}>f_{m}$, that results less computation time for the task.

#### 3.3.3. Platoon Data Transmission

If the task k is executed by the platoon members, the data needs to be offloaded to the destination vehicle terminal before it is executed. The data transmission time for offloading the task k from vehicle $v_{1}$ to vehicle $v_{2}$ is computed by

(9) $$t_{v_{1},v_{2}}^{k}=\frac{d_{k}}{R_{v_{1},v_{2}}^{k}}$$

where $R_{v_{1},v_{2}}^{k}$ is the data transmission rate from vehicle $v_{1}$ to vehicle $v_{2}$. We have

(10) $$R_{v_{1},v_{2}}^{k}=B_{1}\log_{2}\left(1+\frac{\xi_{v}{\mathrm{PL}}_{v}\left(l_{v_{1},v_{2}}\right)}{N_{0}}\right)$$

where $\xi_{v}$ is the signal transmission power of the vehicle, $N_{0}$ is the noise power and $B_{1}$ is the bandwidth for V2V communication. If $v_{1}=v_{2}$, $t_{v_{1},v_{2}}^{k}=0$.

#### 3.3.4. MEC Server Data Transmission

If the task k needs to be offloaded and executed on the MEC server, the data transmission time for offloading the task k from the vehicle $v_{1}$ to the MEC server is computed by

(11) $$t_{v_{1},0}^{k}=\frac{d_{k}}{R_{v_{1},1}^{k}}+\frac{d_{k}}{R_{1,0}^{k}}$$

If the task k needs to be executed on a platoon member, the data transmission time for offloading the task k from the MEC server to the vehicle $v_{2}$ is represented by

(12) $$t_{0,v_{2}}^{k}=\frac{d_{k}}{R_{1,v_{2}}^{k}}+\frac{d_{k}}{R_{0,1}^{k}}$$

$R_{1,0}^{k}$ and $R_{0,1}^{k}$ indicate the transmission data rate from the leader to the MEC server and from the MEC server to the leader respectively. We have

(13) $$R_{1,0}^{k}=B_{2}\log_{2}\left(1+\frac{\xi_{v}{\mathrm{PL}}_{\mathrm{MEC}}\left(l_{1,0}\right)}{N_{0}}\right)$$

and

(14) $$R_{0,1}^{k}=B_{2}\log_{2}\left(1+\frac{\xi_{\mathrm{MEC}}{\mathrm{PL}}_{\mathrm{MEC}}\left(l_{0,1}\right)}{N_{0}}\right)$$

where $\xi_{\mathrm{MEC}}$ is the transmission power of the MEC server and $B_{2}$ is the bandwidth for V2I communication. Because the platoon members cannot communicate directly with the BS, the data needs to be transmitted to the BS through the leader. The connection between BS and MEC is wired, and the time of wired transmission is ignored here.

## 4. Delay Constrained Offloading

**Figure 5:**
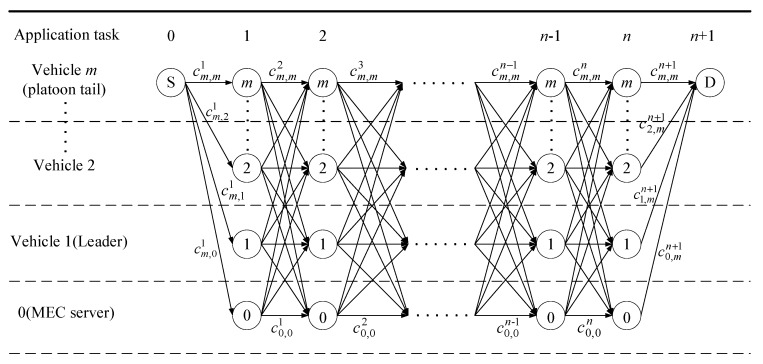
The task execution flow for collaborative offloading decision.

The tasks of an application are represented by the directed acyclic graph G=(V,C). V=0, 1, 2, …, m is the finite node set that represents the task offloading nodes, and C is the edge set that denotes the cost of task offloading. Figure 5 shows the task execution flow for collaborative offloading decision. S is the starting point of the application and D is the task destination terminal. For simplicity, take vehicle m as the requestor of the application as an example. An application contains n+2 tasks. Except for the initial task and the last task, all the platoon members and the MEC server have the opportunity to execute the other n tasks.

Denote x and y are adjacent node numbers of an edge, x, y∈V and x≠y. Each edge corresponds to a task offloading decision and the weights of the edges $c_{x,y}^{k}$ are non-negative value, namely $c_{x,y}^{k}\geq 0$. This weight value stands for the cost of offloading the task and the time it takes to execute the task. Specifically, if the weight value is considered to be a cost price and the task k needs to be offloaded from x to y for execution, we obtain the weight $c_{x,y}^{k}=b_{k}^{y}$. Additionally, if the weight value is considered to be a time delay, we obtain the weight value $c_{x,y}^{k}=t_{k}^{y}+t_{x,y}^{k}$ that is the sum of the computation time and the transmission time of the task k. Therefore, two directed acyclic graphs with respect to the cost price and time delay can be obtained.

Under this framework, we then transform the optimal task offloading decision into a shortest path problem to find the path with minimum cost between nodes S and D. It is constrained by the deadline time ($T_{d}$) of the tasks, and the time delay of the path should be less than or equal to $T_{d}$. If the time delay of a path p satisfies the constrained conditions, p is an appropriate path. A path $p^{*}$ is an optimal path with the minimum cost among all appropriate paths. We mathematically formulate the problem as a constrained shortest path problem by

(15) $$\begin{array}{ll} \underset{p\in P}{\min} & b\left(p\right)=\overset{n}{\underset{k=1}{\sum }}b_{k}^{y}\\ \;s.t. & \left(C1\right):d\left(p\right)=\overset{n+1}{\underset{k=1}{\sum }}\left(t_{x,y}^{k}+t_{k}^{y}\right)\leq T_{d}\\ & \left(C2\right):f_{v_{1}}<f_{0},\;f_{v_{2}}<f_{0} \end{array}$$

where p is the path from node S to node D, and P is the set of all possible paths, p∈P. (C1) is to ensure that the time consumed in executing the tasks is within the deadline. (C2) indicates that any platoon member provides less computational resources than the MEC server. Since each task has m+1 offloading decision options, there are ${\left(m+1\right)}^{n}$ possible options for the offloading strategy.

## 5. Optimal Offloading Decision for Collaborative Task Execution

In this section, we derive an optimal solution for Equation (15) and develop an optimal offloading decision for collaborative task execution. The constrained optimization problem in Equation (15) has been proved to be NP-complete [32].

### 5.1. Optimal Offloading Based on LARAC

It has been previously proposed that the constrained shortest path problem can be solved by LARAC algorithm [33]. We first denote a LARAC function

(16) $$L\left(\lambda \right)=\underset{p\in P}{\min}\left[b_{\lambda }\left(p\right)\right]-\lambda T_{d}$$

where $b_{\lambda }\left(p\right)=b\left(p\right)+\lambda d\left(p\right)$ and λ is the Lagrangian multiplier. By using the Lagrangian duality principle, we can proof $L\left(\lambda \right)\leq b\left(p^{*}\right)$.

Next, we employ Algorithm 1 to find the path with smallest $b_{\lambda }$ between S and D. In Algorithm 1, the *PathAlgorithm* is a process (e.g., Bellman–Ford algorithm [34]) to find the shortest path between S and D with the cost C. The details of the *PathAlgorithm* adopted in this paper will be explained in Section 5.2. If we can find the minimum-cost path under all constraints, this path will be the offloading strategy, otherwise update $p_{d}$ and $p_{b}$ repeatedly to find the optimal λ.

**Algorithm 1.** Find minimum-cost path of $b_{\lambda }$ for collaborative task execution.
**1.    Input:**
**2.**               $S,\;D,\;T_{d}$
**3.**    $p_{b}\leftarrow PathAlgorithm\left(S,\;D,\;b\right)$**4.    If**     $d\left(p_{b}\right)\leq T_{d}$
**then****5.**              return $p_{b}$
**6.    end if**
**7.**    $p_{d}\leftarrow PathAlgorithm\left(S,\;D,\;d\right)$
**8.    If**
$d\left(p_{d}\right)>T_{d}$
**then**
**9.**              return “There is no solution”
**10.    end if**

**11.    while true do**
**12.**              $\lambda \leftarrow \frac{b\left(p_{b}\right)-b\left(p_{d}\right)}{d\left(p_{d}\right)-d\left(p_{b}\right)}$**13.**              $p_{\lambda }\leftarrow PathAlgorithm\left(S,\;D,\;b_{\lambda }\right)$
**14.              If**
$b_{\lambda }\left(p_{\lambda }\right)=b_{\lambda }\left(p_{b}\right)$
**then**
**15.**                    return $p_{d}$
**16.              else**

**17.                    if**
$d\left(p_{\lambda }\right)\leq T_{d}$
**then**
**18.**                       $p_{d}\leftarrow p_{\lambda }$

**19.                    else**
**20.**                       $p_{b}\leftarrow p_{\lambda }$

**21.                    end If**

**22.              end If**

**23.    end while**

**24.    Output:**
$p_{\lambda }^{*}$


The computational complexity of the dynamic programming algorithm depends on the number of nodes, namely O(|V|). In addition, as shown in Reference [35], the Lagrangian multiplier of optimum is obtained after $O\left(\left|N\right|\log^{2}\left|N\right|\right)$ iterations, so the overall computational complexity of the Algorithm 1 is $O\left(\left|V\right|\left|N\right|\log^{2}\left|N\right|\right)$, namely $O\left(n^{2}\log^{2}n\right)$.

Although the algorithm cannot guarantee to find the optimal path, it can obtain a lower bound of the optimal solution. Moreover, its running time is shown to be polynomial [33].

### 5.2. Dynamic Programming Algorithm

In order to apply the Algorithm 1 to the optimal task offloading decision, we need to find the shortest path according to the task execution cost, execution time and aggregation cost. Specifically, we treat all tasks as a multistep process with chain structure, namely a multistep decision process.

**Figure 6:**
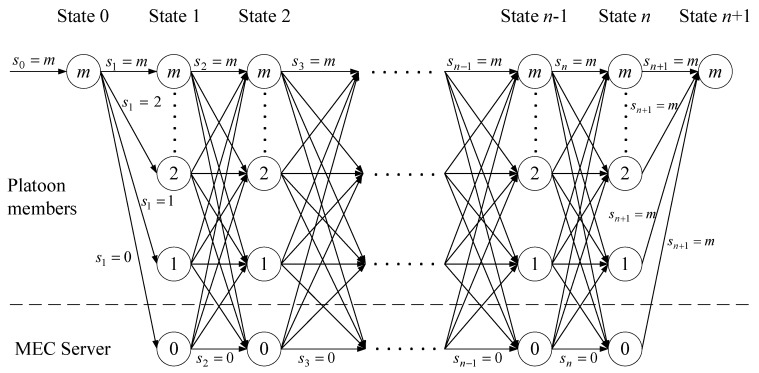
Task state transition process.

The state transition process for the optimal offloading decision of collaborative task execution is depicted in Figure 6. State 0 and state n+1 represent the start and end of the whole application execution respectively.

We denote $r_{k}$ as the location identifier of the task k. For instance, $r_{k}=1$ indicates that the task k is executed on Vehicle 1 in the platoon. $r_{k}$ keeps tracking the position of the application tasks, with 1 to m indicating the platoon members and 0 for the MEC server, as shown in the following

(17) $$r_{k}=\{\begin{array}{ll} 0, & \mathrm{MEC}\;\mathrm{server};\\ 1, & \mathrm{Vehicle}\;1;\\ 2, & \mathrm{Vehicle}\;2;\\ \ldots & \\ m, & \mathrm{Vehicle}\;m. \end{array}$$

Task n+1 is the output result of the application. Since the output result needs to be sent back to the starting point after the completion of task n, it can be obtained that $r_{0}=m$ and $r_{n+1}=m$. The output result of task n+1 does not need to be calculated, and the user does not need to purchase the computing resource, so we have $b_{n+1}^{m}$.

$s_{k}$ is the decision variable for state k, indicating the choice of which vehicle the task k should be offloaded to, shown as follows

(18) $$s_{k}=\{\begin{array}{ll} 0, & \mathrm{MEC}\;\mathrm{server}\;\mathrm{execution};\\ 1, & \mathrm{Vehicle}\;1\;\mathrm{execution};\\ 2, & \mathrm{Vehicle}\;2\;\mathrm{execution};\\ \ldots & \\ m, & \mathrm{Vehicle}\;m\;\mathrm{execution}. \end{array}$$

$s_{0}$ is the task initiation decision and $s_{n+1}$ denotes the last decision of the application, that is $s_{0}=s_{n+1}=m$. The aim is to find an optimal offloading decision strategy set $S^{*}=\left\{s_{0},\;s_{1},\ldots ,\;s_{n+1}\right\}$. 

We next try to find the minimum cost, time delay, and aggregation cost in Figure 6, based on the established iterative equations, respectively.

First, we denote $G_{k-1}\left(r_{k-1}\right)$ as the minimum cost from task k−1 to task n+1. $G_{0}\left(r_{0}\right)$ is the minimum cost for all tasks of the application, given $G_{n+1}\left(r_{n+1}\right)=0$. On this basis, we establish an iterative equation of the latter term with the minimum cost. Knowing $G_{k}\left(r_{k}\right)$ at state k for location $r_{k}$, we obtain every decision at state k−1 so that the cost from state k−1 to state n+1 is minimized. The backward iteration equation with minimum cost is represented by

(19) $$\begin{array}{l} G_{k-1}\left(r_{k-1}\right)=\underset{s_{k}}{\min}\left[b_{k}\left(r_{k-1},s_{k}\right)+G_{k}\left(r_{k}\right)\right]\\ G_{n+1}\left(r_{n+1}\right)=0 \end{array}$$

where $b_{k}\left(r_{k-1},s_{k}\right)$ refers to the cost to be paid for making the offloading decision $s_{k}$ fter task k−1 at position $r_{k-1}$ is completed. Both $G_{k}\left(r_{k}\right)$ and $b_{k}\left(r_{k-1},s_{k}\right)$ are known. The latter one can be obtained using (6) and (8) when the task is offloaded, that is, $b_{k}\left(r_{k-1},s_{k}\right)=b_{k}^{y}$ and $y=s_{k}$. The system state starts from the state n, and the value of the $G_{n}\left(r_{n}\right)$ can be computed from the initial condition $G_{n+1}\left(r_{n+1}\right)$ given in the state n+1. Repeat this process for numerical iteration, and we find the optimal objective function value, optimal decision, and optimal path for the entire multistep decision problem in reverse order.

Second, let $H_{k-1}\left(r_{k-1}\right)$ denote the minimum completion time from task k−1 to task n+1. $H_{0}\left(r_{0}\right)$ is the minimum task completion time for all tasks of the application, given $H_{n+1}\left(r_{n+1}\right)=0$. Knowing $H_{k}\left(r_{k}\right)$ at state k for location $r_{k}$, we obtain every decision at state k−1 so that the time delay from state k−1 to state n+1 is minimized. The backward iteration equation with minimum task completion time is expressed by

(20) $$\begin{array}{l} H_{k-1}\left(r_{k-1}\right)=\underset{s_{k}}{\min}\left[t_{k}\left(r_{k-1},s_{k}\right)+H_{k}\left(r_{k}\right)\right]\\ H_{n+1}\left(r_{n+1}\right)=0 \end{array}$$

where $t_{k}\left(r_{k-1},s_{k}\right)$ refers to the completion time of task k being offloaded to $s_{k}$ after task k−1 at position $r_{k-1}$ is executed. Both $H_{k}\left(r_{k}\right)$ and $t_{k}\left(r_{k-1},s_{k}\right)$ are known. The latter one can be obtained using (5), (7), (9) and (11) when the task is offloaded, that is, $t_{k}\left(r_{k-1},s_{k}\right)=t_{x,y}^{k}+t_{k}^{y}$ and $y=s_{k}$.

Third, let $J_{k-1}\left(r_{k-1}\right)$ be the minimum aggregated cost from task k−1 to task n+1. $J_{0}\left(r_{0}\right)$ is the minimum aggregated cost for all tasks of the application, given $J_{n+1}\left(r_{n+1}\right)=0$. Knowing $H_{k}\left(r_{k}\right)$ at state k for location $r_{k}$, we obtain every decision at state k−1 so that the aggregated cost from state k−1 to state n+1 is minimized. The backward iteration equation with minimum task aggregated cost is represented by

(21) $$\begin{array}{l} J_{k-1}\left(r_{k-1}\right)=\underset{s_{k}}{\min}\left[b_{k}\left(r_{k-1},s_{k}\right)+\lambda t_{k}\left(r_{k-1},s_{k}\right)+J_{k}\left(r_{k}\right)\right]\\ J_{n+1}\left(r_{n+1}\right)=0 \end{array}$$

We use iterative Equations (19)–(21) to implement the processes of *PathAlgorithm**(S, D, b)***, *PathAlgorithm**(S, D, d)*** and *PathAlgorithm**(S, D, b_λ_)*** to find the minimum cost, time delay and aggregated cost, respectively. Finally, the minimum-cost offloading decision strategy with time delay constraints is obtained in Algorithm 1.

The proposed approach is designed for the applications considered. This is not the only scenario that can be used. Other scenarios, such as an individual vehicle or a cluster of vehicles in the cellular network, also can use the proposed method.

## 6. Numerical Analysis

In this section, the performance of the proposed task offloading decision strategies for an MEC-assisted platoon is evaluated.

### 6.1. Application Profile

We evaluate the efficiency of our algorithm in a cellular network composed of a BS with an MEC server. The distance between the leader and BS is η=0.5 km. The distance between the adjacent vehicular transmitter and the receiver is μ=0.008 km. The platoon consists of nine members (i.e., m=9) and the distance between vehicles is fixed in the platoon. Assume that the antenna position of each vehicle is the same. Assume that the antenna position of each vehicle is the same. The communication mode is deployed in accordance with the relevant rules of IEEE 802.11p standard and LTE-V. The bandwidth and transmission power for V2V and V2I communications in the simulation are set according to the reference papers [31,36]. The parameters of computation resource are set as follows: $f_{0}=3000\;\mathrm{MHz}$, $f_{1}=100\;\mathrm{MHz}$, $f_{2}=650\;\mathrm{MHz}$, $f_{3}=600\;\mathrm{MHz}$, $f_{4}=620\;\mathrm{MHz}$, $f_{5}=700\;\mathrm{MHz}$, $f_{6}=800\;\mathrm{MHz}$, $f_{7}=660\;\mathrm{MHz}$, $f_{8}=1000\;\mathrm{MHz}$, $f_{9}=550\;\mathrm{MHz}$. The other parameters in the simulation are summarized in Table 1.

### 6.2. Minimum-Cost Decision Strategy

**Figure 7:**
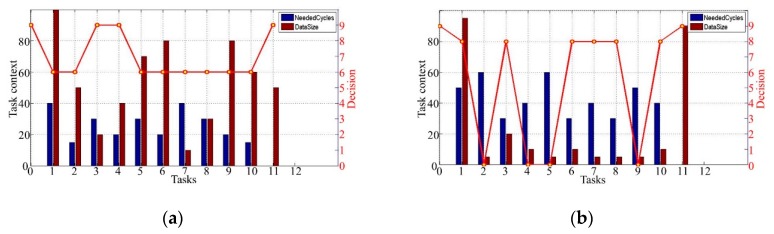

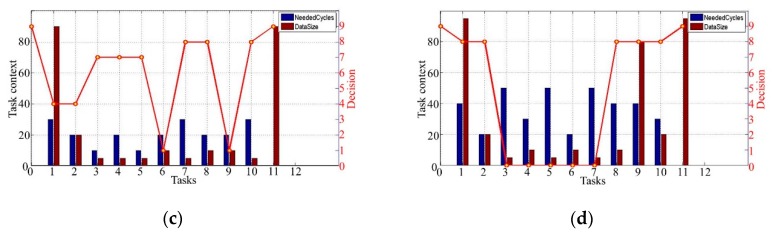
Task offloading decisions. (**a**) low computing cycles with high size of data; (**b**) high computing cycles with low size of data; (**c**) low computing cycles with low size of data; (**d**) high computing cycles with high and low size of data.

We consider a mobile application that consists of 12 tasks. The deadline of the application is 0.3 s, the computation resource cost of MEC server is $\alpha_{\mathrm{MEC}}=0.9$ and the offloading decisions in detail are shown in Figure 7. In these four figures, the red line represents the tasks offloading decision $S^{*}$, which is the shortest path with the lowest cost and within the deadline. The NeededCycles means the computing workload. The task context includes two parts: the NeededCycles, whose unit is cycle, and the DataSize, whose unit is Kilobyte (kb). Figure 7 indicates different offloading strategies based on the proposed algorithm for tasks with different computing workloads and data sizes. Specifically, in Figure 7a, the amount of computation cycles required for each task of the application is relatively small and transmission data size of the tasks is relatively large. We get $S^{*}=\left\{9,6,6,9,9,6,6,6,6,6,6,9\right\}$, because the transmission data rate between vehicles is much faster than that between leader and BS, and the large data size of the tasks limits the task execution on the MEC server. If the task is to be offloaded to the MEC server, it will lead to a large transmission delay. In Figure 7b, $S^{*}=\left\{9,8,0,8,0,0,8,8,8,0,8,9\right\}$. Note that the size of the transmitted data is relatively small, and the required computation cycles for each task increase. In order to satisfy the deadline constraint of the application, a task with high required computation cycles should be offloaded to the MEC server. In Figure 7c, $S^{*}=\left\{9,4,4,7,7,7,1,8,8,1,8,9\right\}$. Each task requires a relatively small amount of computation cycles. To minimize the cost, the MEC server with higher computing power does not need to provide computational assistance to the task execution. In Figure 7d, $S^{*}=\left\{9,\;8,8,0,0,0,0,0,8,8,8,9\right\}$. Note that last four tasks are executed in the platoon to avoid high data transmission delays from the BS to the leader. These four cases show that the MEC server and the BS can cooperate to complete the application tasks. 

Figure 7 indicates that the computing workloads and the data size affect the offloading decisions. A task with low computing cycles and high size of data tends to be executed in the platoon instead of MEC server, because the impact of task transmission time is greater than that of computing time, and these kinds of tasks are more likely to be offloaded to the nodes with fewer computing resources for lower cost. On the contrary, a task with high computing cycles and low size of data tends to be executed at the nodes with more computing resources in the platoon or at the MEC server decided by the cost and deadline. Note that the tasks with high computing cycles and low size of data is offloaded to the MEC server, which do not always meet the requirements of system. For instance, if the penultimate task with high computing cycles and low size of data was offloaded to the MEC server to execute, and the last task with 0 computing cycle and high size of data must be executed at the platoon member. That will cause extremely large transmission time when the data of last task is transmitted back to the member from the BS so that execution time of all tasks may exceed the deadline. Our algorithm succeeds in avoiding this. In summary, the LARAC algorithm can offload the tasks reasonably, and the task execution deadline can be satisfied.

**Figure 8:**
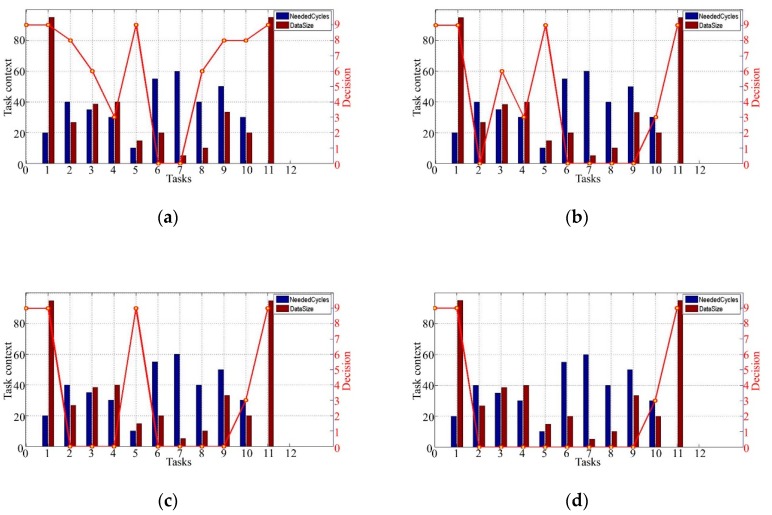
Task offloading decisions. (**a**) $\alpha_{\mathrm{MEC}}=0.4$; (**b**) $\alpha_{\mathrm{MEC}}=0.3$; (**c**) $\alpha_{\mathrm{MEC}}=0.2$; (**d**) $\alpha_{\mathrm{MEC}}=0.1$.

We next evaluate the effect of *α_MEC_* on the tasks offloading decisions. The NeededCycles and the DataSize of these four subfigures in Figure 8 are the same. In Figure 8a, the cost of offloading the tasks to the MEC server is greater than that of any member of the platoon. To meet the deadline of the application, Tasks 6 and 7 must be offloaded to the MEC server for shorter execution time. In Figure 8b, we can see that, besides Tasks 6 and 7, Tasks 2, 8, and 9 are offloaded to the MEC server to execute. Similarly, in Figure 8c,d, we can see that more tasks are offloaded to the MEC server compared with that in Figure 8a. This is because, as α_MEC_ getting smaller, more of the tasks are offloaded to the MEC server for saving the cost. The proposed algorithm in this paper considers the tasks offloading strategy with minimum cost. As α_MEC_ becomes smaller, the cost of offloading tasks to the MEC server decreases gradually, so that more tasks will be offloaded to the MEC server.

### 6.3. Comparison of Execution Patterns

**Figure 9:**
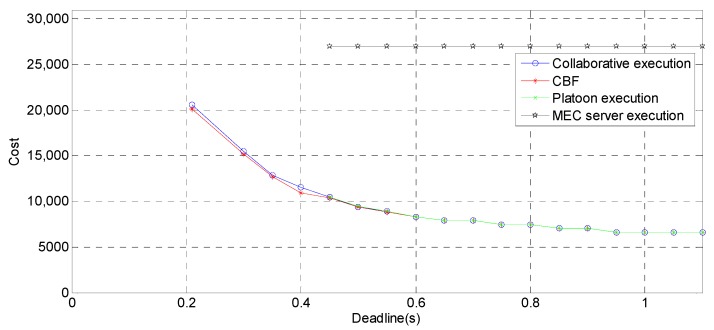
Deadline vs. cost.

Figure 9 shows the cost comparison of three execution modes (platoon execution, MEC execution and proposed collaborative execution) and the Constrained Bellman–Ford (CBF) algorithm [37], $\alpha_{\mathrm{MEC}}=0.4$. The CBF algorithm could also be adopted to solve this problem, but its computational complexity is $O\left(n^{3}\right)$, which is larger than $O\left(n^{2}\log^{2}n\right)$ in this paper. Additionally, the CBF algorithm gets the optimal solution, while the LARAC algorithm gets the approximate solution. The data sizes and the demanded computation cycles are as follows [19]: $\left\{d_{k}\right\}=\left\{0\;100\;40\;1\;2\;1\;2\;1\;2\;1\;1\;100\right\}$ kb,  Mcycle. First, compared with MEC server execution, the proposed collaborative execution strategy can greatly reduce the cost. Second, collaborative task execution is more flexible than MEC server execution. Due to the low transmission rate between the leader and the BS, the high transmission delay will be caused by the MEC server execution when the data size is large. Therefore, the tasks with a large amount of transmitted data cannot be completed within the deadline in MEC server execution mode. Third, only collaborative task execution can complete the application less than the deadline 0.21 s, and it costs less than that in the MEC server execution mode. Fourth, with increase of the deadline, the cost of platoon task execution and collaborative task execution are approximately same, because the task execution does not require the participation of MEC server with the release of the deadline. The computational resources provided in the platoon are sufficient to enable the application to be completed within the deadline. The results of the local execution were not drawn, because 3.3 s is the minimum deadline, far from meeting the requirements of the application time constrained. Fifth, we can see that the cost of the collaborative task execution is very close to that of the CBF algorithm, which verifies the proposed algorithm can be well applied in this scenario.

## 7. Conclusions and Future Works

In this paper, the procedure of cooperative tasks execution for an MEC-assisted platoon within the execution deadline is studied. The task offloading decision problem is transformed into the shortest path problem in a directed acyclic graph. We employ the “LARAC” algorithm to obtain the optimal decision strategy for task offloading. The results show that there exists more than one migration between the platoon members and the MEC server, and all the members have the opportunity to participate in the tasks execution. Moreover, the proposed collaborative task execution strategy can greatly reduce task execution cost and execution time. 

For our future work, we will consider tasks offloading for a more general case of vehicular network, such as a cluster of vehicles in a cellular network. In addition, tasks offloading of crossing cell—which depends on the deployments of the MEC severs—will be achieved in future discussion.

## Figures and Tables

**Table 1:** Simulation parameters.

Parameters	Value
The number of platoon members	9
Bandwidth for V2V communication	$B_{1}=20\;\mathrm{MHz}$
Bandwidth for V2I communication	$B_{2}=100\;\mathrm{MHz}$
The noise power	$N_{0}=-174\;\mathrm{dBm}/\mathrm{Hz}$
The transmission power of vehicle	$\xi_{v}=23\;\mathrm{dBm}$
The transmission power of BS	$\xi_{\mathrm{MEC}}=46\;\mathrm{dBm}$
The distance between the adjacent transmitter and the receiver	μ=0.008 km
The distance between the leader and BS	η=0.5
the computation resource cost of vehicle	$\alpha_{v}=1$
